# Travelling along a road with obstacles: Experiences of managing life to feel well while living with migraine

**DOI:** 10.3402/qhw.v8i0.19900

**Published:** 2013-02-05

**Authors:** Stina Rutberg, Kerstin Öhrling, Catrine Kostenius

**Affiliations:** Department of Health Sciences, Luleå University of Technology, Luleå, Sweden

**Keywords:** Hermeneutic-phenomenology, headache management, lived experiences, patient perspective, health promotion, well-being

## Abstract

Living a life with migraine can impair one's sense of feeling well, and migraine is a disorder that is associated with substantial disability. Earlier research on how people manage their migraine has given important insight into these people's preventive actions and how they handle their attacks, but there is still a lack of knowledge of how persons with migraine manage their lives to feel well from a more holistic viewpoint. Therefore, the aim of this study was to explore lived experiences of managing life to feel well while living with migraine. Nineteen persons with migraine were interviewed. A hermeneutic-phenomenological approach was used to explore their lived experiences. The findings reveal that persons with migraine not only used preventive strategies to abort and ease the consequences of migraine but also tried to amplify the good in life through increasing their energy and joy and through reaching peace with being afflicted with migraine. The findings of this study can encourage healthcare providers, as well as persons with migraine, to consider channeling their efforts into strategies aiming to amplify the good in life, including reaching peace of mind despite being afflicted.

Living with migraine means having recurrent painful headaches, often associated with symptoms such as nausea, photophobia, or phonophobia (Blumenfeld et al. 2011). However, the periods between the attacks are not symptom-free, and quite often the person with migraine experiences fear or worry about the next attack (Wacogne, Lacoste, Guillibert, Hugues, & Le Jeunne, 2003). Thus, his or her life could revolve around maintaining readiness for an attack and making arrangements to avoid triggering migraine (Rutberg & Öhrling, 2012).

Migraine is a chronic disorder that is associated with substantial disability (Leonardi, Raggi, Ajovalasit, Bussone, & D'Amico, 2010), so living with migraine can impair the quality of life (Brandes, 2008). To improve their health, persons with migraine actively involve themselves in the management of their disorder (Peters, Abu-Saad, Vydelingum, Dowson & Murphy, 2003). While researching the ways that persons with migraine manage their disorder, Peters, Abu-Saad, Vydelingum, Dowson, and Murphy (2004) discovered that these people rely on a problem-focused management that comprises seeking information and support and taking problem-solving actions. In a previous study, Peters et al. (2003) also found that people with migraine operated on the basis of a justification and consequence system, for both acute and preventive management of migraine. Moreover, both Moloney, Strickland, DeRossett, Melby, and Dietrich (2006) and Meyer (2002) concluded that the women they interviewed were always prepared to prevent and control their migraine. The research thus far on managing migraine gives important insight into how persons with migraine try to control and abort their migraine attacks; however, there is still a lack of knowledge of how persons with migraine manage their lives to feel well in addition to taking preventive measures. Thus, we agree with Anand and Sharma (2007) that more qualitative research is needed about the management of migraine from the patients’ perspective because such research could provide important information for doctors and other healthcare professionals, and possibly help practitioners to develop future care and education.

Good health is more than only the absence of disease or weakness. In 1947, the World Health Organization defined good health as a state of complete physical, psychological, and social well-being. There is no generally accepted definition of well-being, even though it is often used synonymously with good health (Svensson & Hallberg, 2011). According to Lehnert, Sudeck, and Conzelmann (2012), subjective well-being consists of psychological well-being, both cognitive and affective; somatic well-being, both physical self-evaluations and physical functioning; and social well-being. We agree with the view that the concept of well-being rests on an implicit view of what it means to be a whole and healthy human being, and that it is important to strive to look at the whole picture of how persons with migraine manage their lives to feel well (Sarvimäki, 2006). Therefore, the aim of this study is to explore lived experiences of managing life to feel well while living with migraine.

## Methodological framework

The lifeworld is the world that we all participate in and are a part of, and it is as well the world of lived experience (Husserl, 1970). The framework of this study is the lifeworld, as it is through the lived experiences that researchers, using a hermeneutic-phenomenological approach, search for knowledge. To understand the meaning of a phenomenon, we need to interpret the lived experience of the phenomenon, and according to van Manen (1997), phenomenology means understanding being in the lifeworld. In this study, we are interested in knowing more about how it is to be in the world as a person living with migraine and managing life to feel well.

According to Gadamer (1994), the human being can only understand the world on the basis of earlier experiences, theories, or philosophies. He further argues that researchers try to fuse their horizon of understanding with the horizon provided by participant in studies or by text to increase knowledge. Agreeing with Gadamer (1994) and van Manen (1997) that it is not possible to suspend or bracket one's preunderstanding and beliefs, the present authors have tried to make them explicit, in order to meet the phenomenon in a way that is as free and unprejudiced as possible. None of the present authors has experience of living with migraine or other chronic disorders. However, that does not free us from presumptions of what it means to manage life to feel well while living with migraine. Previously, we conducted a study of the meaning of living with migraine (Rutberg & Öhrling, 2012), and we have worked clinically (SR as a physical therapist, KÖ as a nurse, and CK as a health consultant) and met people with chronic disorders trying to improve their health.

## Method

### Participants and procedure

Seventeen women and two men diagnosed with migraine participated in the study. The criteria for participation were a minimum age of 18 years, living with migraine and the ability to speak Swedish. The participants’ age ranged from 20 to 69 years (md: 48 years), and 17 persons had been experiencing their symptoms for about 9–59 years, while two persons had had their symptoms and diagnosis for about 1 year or less. The number of migraine attacks varied: three participants experienced 1–8 attacks per year, 10 participants experienced 1–4 attacks per month, four experienced 5–8 attacks per month, and two experienced 12–25 attacks per month. Eleven persons were working or studying full-time, and three persons were working or studying part-time due to migraine. Four persons were pensioners, and one was receiving a disability pension.

The participation in the study was arranged through the Swedish Migraine Association, as well as through physical therapists at primary-care clinics in the northern part of Sweden. The Swedish Migraine Association forwarded letters describing the purpose of the study to all its members in the northern part of Sweden. Eight persons answered, saying that they would be interested in participating in the study. The physical therapists at the primary-care clinics were contacted by phone and asked if they were presently treating any persons diagnosed with migraine. At nine of those clinics, the physical therapists were treating at least one person with migraine. We sent information about the study to the physical therapists at each clinic, and they passed the information to their patient(s). The persons interested in participating were contacted by phone, and additional information about the study was given and the time and place of an interview were decided. Twelve interviews took place at the home or workplace of the participant, and seven interviews were conducted at different campuses at the Luleå University of Technology, all of them held in an undisturbed room.

### Data collection

This study is built on in-depth interviews with a narrative approach. The interviews was conducted by the first author and digitally recorded. A pilot interview was performed to test the research questions; then, minor revisions were made. The participants were asked to talk about their experiences of managing their life with migraine and what they did to feel well. To encourage the participants to elaborate upon their experiences, the interviewer asked questions like, “How did you feel then?” and “Can you tell me more about …?” The interviews lasted between 35 and 135 minutes each and were transcribed verbatim.

### Data analysis

To transform personal meanings and experiences from interview text into understanding, we chose to use a hermeneutic-phenomenological approach inspired by van Manen (1997). The analysis was guided by two overarching questions: “What makes this lived experience what it is?” and “What is unique about this experience?” (van Manen, 1997). Hermeneutic-phenomenological research follows interrelated phases of seeking meaning, theme analysis, and interpretation with reflection activities that are performed in a movement back and forth to reach understanding of the phenomenon (van Manen, 1997).

Each interview was read and reread to capture the main significance of the interview as a whole, inspired by van Manens (1997) “wholistic approach” (p. 93). Aspects or qualities of the phenomenon were written down as essential themes. To find the characteristics of the essential themes, the interviews were read again line-by-line, and statements or phrases that seemed to reveal aspects of lived experience of managing life to feel well were highlighted. van Manen (1997) refers this process to a selective and detailed approach. To do justice to the fullness and ambiguity of the participants’ experiences of managing life with migraine, we involved ourselves in a process of writing and rewriting. van Manen (1997) argues that rewriting is a complex process of rethinking, reflecting, and recognizing; he explains, “writing teaches us what we know and in what way we know what we know” (p. 127). Therefore, writing is a way to exercise self-consciousness. We discussed the essential themes and their characteristics throughout the analysis process and then presented our findings as a whole through two overarching themes and four subthemes.

The process of making our preunderstanding, assumptions, and beliefs as explicit as possible started early with discussions about our perceptions of well-being, migraine, and managing life to feel well, and we kept a record of our preunderstanding. Inspired by Drew (2001), we tried to explore our preunderstanding by writing personal statements about the highlighted phrases that we had chosen to reveal aspects of managing life to feel well. We then attempted to find the questions that the personal statements potentially answered, to become aware of our preunderstandings and what we were overlooking. To challenge further our understanding of the preliminary findings, we discussed them with researchers who were highly experienced in qualitative research methods and healthcare.

### Ethical considerations

The Regional Ethical Review Board at Umeå in Sweden gave their approval of this study (Ref. no. 08–182M). The persons included in the study gave their informed consent to participate after full confidentiality was assured and after they received information that they could change their mind about participating at any time without providing any explanation.

## Findings

The analysis revealed two main themes and four subthemes, which capture the experiences of managing life to feel well while living with migraine (Table I).

**Table I:** Overview of the main themes and subthemes.

Main theme	Building a foundation of safeness
Subtheme	Using experiences of triggers as a guide
Subtheme	Striving for power to be in control
	
Main theme	Amplifying the good in life
Subtheme	Acting thoughtfully to increase one's energy and joy
Subtheme	Being in a process of accepting migraine as a part of life

### Building a foundation of safeness

To feel well while living with migraine, it was necessary to try to avert the threat of having a migraine attack, as well as to possess potent methods of alleviating the attacks. The persons used their experiences of triggers as a guide, to increase their sense of control over migraine. Their experiences of migraine attacks influenced their way of managing triggers. Being able to alleviate the attacks induced a sense of safeness and gave them a sense of increased power, enabling them to take control of their lives. This feeling of safeness made it possible for them to experiment with triggers and to explore new possibilities of feeling well. Being attentive to their migraine increased their knowledge and helped them to build a foundation of safeness.

#### Using experiences of triggers as a guide

To reduce the risk of having a migraine attack, the persons with migraine constantly searched for and had become aware of triggers. In their search for triggers, they also considered other people's experiences of triggers through sharing them in conversations or by reading about them in magazines. It was important to take all the triggers into consideration, as it was sometimes possible to expose oneself to one trigger, but not to a combination of several triggers. In addition, the persons with migraine experienced different degrees of sensitivity to triggers. They furthermore experienced that their personal energy levels affected their sensitivity, so they considered it important not to let their level of energy decrease, in order to avoid triggering migraine. These experiences had led them to eat at regular times, to cherish a good night's sleep, and to carry out activities that increased their energy.

I know I need to sleep to have the energy … I like to take a glass of red wine or a drink, but I know that in combination with stress and other things, it is easier for me to get a migraine; then you learn to balance; if you have had a stressful week or slept badly or other things in life that affect you, then you do not drink a glass of wine on a Friday evening.

Their earlier experiences had taught them how to manage and acquire an awareness of their triggers. The triggers were experienced as being of a varied nature. Some of the triggers of migraine lay beyond the person's control, such as weather and hormonal changes, whereas others were more controllable in the manner that allergens are controllable, for example, triggers such as food, alcohol, and sunlight. To manage triggers, it was important always to be prepared and to appraise whether it was worthwhile exposing oneself to them or not.

… yes, you become a bit like that [attentive], like the sun, because when it is really bright, then you need to use sunglasses, yes, you do not need to provoke fate … all the time you think preventively …”

Triggers like stress had made some of the persons with migraine plan their lives in such a way that they would avoid stress, and others had developed strategies to decrease their levels of stress, such as exercising, practicing relaxation training, and so on.

“… the exercise really helps, it minimizes the attacks … if I am stressed or frustrated, it [the migraine] usually comes, or if I eat badly … the exercise makes me more balanced as a person, more tolerant of stress …”.

Having pain in other body parts, especially the neck or head, increased their proneness to migraine attacks. Those who experienced such pain used exercise and stretching, and some consulted a physical therapist or some other healthcare professional to improve their health. By being attentive and listening to the signals from their bodies, the persons with migraine had found it possible to recognize early signs of triggers. This made it possible to know, for example, when it was necessary to lower their stress or to reduce the strain on the muscles in their neck and shoulders.

#### Striving for power to be in control

One need that people with migraine have is access to potent ways of treating the symptoms of a migraine attack so that they can function. The persons with migraine expressed how they struggled or had struggled previously to discover ways of dealing with the pain and the other symptoms of a migraine attack, and how they had panicked just at the thought of having an attack. Therefore, as long as a migraine attack meant a threat of not being able to function, it imposed an uncertainty in life, and it was vital to have a strategy for acute treatment. Having the possibility of alleviating the symptoms of an attack was a prerequisite for enhancing their sense of being in control of their own lives and, thereby, for increasing their notion of feeling well. With this security, they could make time-sensitive plans, such as holiday travel, parties, and other social events.

The medications are the thing that provides a base of safety … when I have an episode I can take a pill and then I do not need to live in uncertainty; it gives me the power to rule over my life …

To ease the symptoms of migraine, the persons talked mainly about using medication. However, they generally did not want to use more medication than was absolutely necessary, as they were afraid that long-term use could lower the effect of the medication, negatively affect them, or increase the risk of becoming addicted to the medication. This imposed the threat of losing the ability to alleviate the attacks and the power to be in control of one's life. Therefore, they often negotiated the use of medication with themselves, deliberating over what kind of treatment they needed to use and whether to start treating a migraine attack at once, or whether it was possible to wait. Sometimes the easiest way to function quickly was to take medication immediately.

Having preventive strategies could increase the feeling of control over migraine, and some persons took preventive medication or acupuncture treatment to ease their migraine. Avoiding triggers was often considered to be a good and sometimes necessary strategy to avoid migraine, but at the same time, the persons with migraine had to create a balance whereby avoidance did not diminish and limit their lives too much. By experimenting with their triggers and testing their limits, some of the interviewed persons successfully increased their sense of being in control of their own lives.

Every year I try to think of something that frightens me and I challenge myself to do it … last year, I decided to take my dog to an exhibition myself, which does not sound so tough, but when you have migraine, you have a tenseness; I got some attacks a couple of times at these dog exhibitions, but in the end, I managed it and it has gone really well … to me, everything is possible, and that is a really nice feeling.

Moreover, by compensating for the limitations due to avoidance with other activities or things, the sense of having the power to rule one's life could be enhanced. However, persons who did not have potent ways of treating their migraine attacks had a greater need to focus on avoiding triggers. Being in that kind of situation meant that thoughts of migraine occupied a large space in their lives; it limited their activities and ruled over the choices that they could make.

… earlier, then, my energy went to trying to control so that you wouldn't get a migraine; yes everything, all your power went to these things; you did not do anything else … today, I look for things to make me feel better …

### Amplifying the good in life

When the persons in the study experienced a high sense of well-being, they felt increased tolerance of migraine attacks. To increase their well-being, they tried to amplify the good in life by making thoughtful choices. When they had reached a sense of peace through acceptance of the fact that migraine was a part of life, this served as a plateau from which they could find more ways of amplifying the good in life.

#### Acting thoughtfully to increase one's energy and joy

The persons with migraine general sense of well-being affected both the quantity and the severity of their migraine attacks, as well as their ability to handle the concurrent pain and other symptoms. With an increased sense of well-being, migraine attacks were easier to bear, and the number of migraine attacks sometimes could decrease. Conversely, when they were suffering from depressions or facing crises in life, such as a divorce, an attack could be harder to tolerate, and the number of migraine attack could escalate. The persons pursued activities that strengthened their sense of energy and well-being to increase their resistance to migraine. Exercising was a common way to increase one's sense of well-being. In addition to decreasing muscle tensions, lowering stress, and leaving a pleasant feeling in one's body, it instilled the feeling of being capable and having physical strength.

You need to find these things [things that give energy], otherwise it is as if you just bury yourself in thoughts of, ‘I can't do this, then I get pain,’ and then you feel really bad; then you do not find any sunshine in your life … having high energy and feeling well makes me not get so ill …

The persons with migraine made an effort to use their energy and resources wisely, and they found it vital to find the things that increased their sense of health and to be aware of the things that “drained” their energy. They felt that they had become more attentive to what was best for them, such as prioritizing something that reinforced their energy after an attack, instead of hastening to do all the things that they had not managed to do during the attack.

… it [migraine] takes one's energy … I try to enjoy myself [when I do not have migraine] and not just hurry up and clean and wash, etc.; there are always things that are postponed and not prioritized, there is always a lot to do, but you need to rest and think, ‘Oh, how I feel good.’

Nevertheless, when the persons with migraine felt guilty about not being able to fulfill their commitments during an attack, they prioritized the fulfillment of commitments over feeling better. In the long run, however, pushing themselves to meet their own demands and those of others without sufficient time or energy led to them feeling run-down, which could cause them to lose the joy of doing the things that they had appreciated earlier. In contrast, when they took the time to evaluate their resources and energy and prioritized activities of their own choice in addition to obligations, they experienced a better quality of life. Moreover, the persons with migraine told about feeling more joy and self-esteem after they focused on their possibilities rather than their barriers (i.e., limitations due to migraine). Some persons had tried cognitive behavioral therapy or mindfulness and found these approaches to be helpful. By being in the present and trying to enjoy life, they made their lives richer, and worrying about having a migraine attack did not drain as much energy as previously.

When I am healthy and do not have a headache, I think that nobody is happier than me, I really appreciate those days … instead of going around and being bitter or in a bad mood and whining all the time, I have chosen to make the best of the situation; things are fine after all … the days you are sick, you take things for what they are and when you are fine, things are really fine; that is my way of looking at it …

#### Being in a process of accepting migraine as a part of life

Acceptance of being afflicted with migraine had different meanings among the persons with migraine. Those who felt at peace with being afflicted could look back and see that accepting the disorder had been a process of reaching a sense of calm and a security within themselves. This kind of acceptance had its roots in facing the fact that migraine was a part of life that would not go away for a long time or at all. Feeling at peace with being afflicted meant that guilt in general was not present or did not rule them as it had done before. Similarly, they expressed having reduced feelings of remorse for not having the strength and energy to handle things during an attack. Furthermore, it meant that they did not bother as much as they had previously about what everybody else thought and that they found it easier to tell others about their migraine, without being bothered by feelings of embarrassment or shame.

I think it [having acceptance] is important; earlier, I could see myself as a failure when I did not have the energy to do certain things, but now that's the way it is and you need to accept it; if you don't have the strength you don't have it …

There were also persons with migraine who described their acceptance of the disorder as merely knowing the fact that they suffered from it, and they expressed frustration and limitations due to migraine in their life. It was difficult for them to accept the uncertainty of having attacks, and they often compared their lives to the “normal” lives of others or to the time before they were afflicted with migraine. They were more often waiting for the migraine to disappear and dreaming about what life would be like without migraine. Reaching acceptance of migraine as being a part of life and only a part, made it easier to channel efforts into feeling well and not just trying to avert the sources of harm.

I do not have a choice [but to accept it]; it [migraine] is nothing I can opt out of, so I wait for better medications and more research to come so they can arrive at a way for me to get rid of this and so I do not need to have this worry all the time …

Moreover, the persons’ surrounding environment influenced his or her process of acceptance and feeling at peace with being afflicted. The interviewed persons’ inner sense of acceptance fluctuated to some extent depending on the situation and the attitudes of the surrounding people. When the persons with migraine sensed that they were trusted and understood, they found it easier to accept their disorder and to handle the emotions aroused by being afflicted. However, when they encountered people who questioned the seriousness of their migraine and who imposed feelings of guilt when they were unable to perform their duties due to migraine, they found it more challenging to accept their situation. Close relatives and friends, as well as influential people, such as employers, healthcare personnel, and perhaps, in particular, doctors, influenced the persons’ own acceptance of being afflicted with migraine, by either taking them seriously or by causing them to feel mistrusted or dismissed.

I have a good back-up, a doctor who understands: she has migraine herself and when I explain something she nods and recognizes, that is important … I do not care a lot about what other people think, because I know and I am able and I have a great doctor who supports me … she takes me seriously when I tell her that I am in real pain … that migraine is a real sickness …

Fairly often, persons with migraine were asked to explain the reasons why a migraine attack had appeared. For example, they were asked whether they had done or not done this or that, and it was easy for them to perceive such questions as implying that they themselves were responsible for the attack. Therefore, it was important for them to have explanations to give to others as to why they had suffered a migraine attack. Another help in reaching acceptance was the realization that they were neither alone nor strange, which could be achieved, for example, by reading about or meeting other people afflicted with migraine.

Many want to know, ‘What have you eaten?’ I have even heard, ‘Perhaps you laughed too much, so you got tense,’… I know they mean well, but there are hundreds of reasons; it is as if I am responsible, almost as if they mean, ‘What have you done?’… then, I become like, ‘It's none of your business!’ and then I can say that the weather has changed and then there is nothing more to think about … I have accepted, it is me …

## Discussion

The findings of the present study reveal that managing life to feel well while living with migraine means both building a foundation of safeness and amplifying the good in life. Earlier studies on how persons manage their migraine has focused on preventive strategies (Meyer, 2002; Moloney et al. 2006; Peters et al. 2003, 2004). The necessity of promoting the good in life, in addition to preventive strategies in order to feel well, is the finding of the present study that add to previous knowledge. During the analysis, the following parable was written about our understanding:

Managing life with migraine is like travelling along a road with obstacles (i.e., triggers of migraine). In order to avoid harm and to feel safe, you either avoid the obstacles or try to remove them from the road. Hitting one obstacle might be tolerable, but certainly not hitting several. The more one feels that one can handle a hit (alleviate an attacks); the smaller the obstacles on the road appear. By investing in increasing one's energy and power (i.e., increasing one's well-being), one decreases the extent to which one feels hurt by a hit. By accepting the obstacles along the road and the possibility of hitting them as a natural part of the journey, the ride becomes easier and more effort can be channeled into making it as pleasant as possible.

The present study has shown that it is crucial to build a basic foundation of safeness. A means to reach safeness was to avert the threat of migraine, and the persons in the present study talked about dealing with triggers. However, the interpretations that some triggers lower the energy levels of the person with migraine and that having decreased energy would, therefore, increase the risk of having a migraine attack are not to be found in previous literature and must, therefore, be treated with caution. Moreover, Brandes (2008) has stated that migraine affects well-being, but whether well-being affects migraine is not so evident in previous research. The findings in the present study point to increased well-being giving higher tolerance of migraine, and the persons with migraine describe how they tried to amplify the good in life as a strategy to feel better. This is in line with Varkey, Linde, and Henoch (2012) who found that persons with migraine use strategies to enhance their well-being and that the sense of feeling well reduced the burden associated with migraine. Therefore, we argue that, in the treatment of migraine, efforts should be channeled into increasing the energy and well-being of persons with migraine to raise their migraine threshold and decrease their burden of migraine, instead of just using preventing strategies.

The persons with migraine used their experience of triggers as a guide to avoid migraine. Triggers were like obstacles on the road of life, which they sometimes were able to avoid and sometimes needed to tackle. When the avoidance restricted life, particularly with regard to social relations or physical activity, it was hard to bear. Recent research (Martin, 2010; Martin & MacLeod, 2009) has questioned the non-evidence-based, yet commonly accepted, preventive strategy of avoiding migraine triggers. Instead, they argue in favor of learning to cope with triggers as a more effective long-term strategy. In the present study, some persons with migraine had tried to challenge the limitations imposed by triggers and found that this enriched their lives, enabling them to pursue more activities. However, persons who sensed that they lacked potent ways of alleviating an attack expressed no willingness to experiment with triggers, as the symptoms of the attack were experienced as worse than the limitations of avoidance.

Possessing potent ways of alleviating attacks was understood as increasing one's power to be in control of one's life, by making it possible to function during an attack. It is possible to draw parallels between the present finding and the findings of Heath, Saliba, Mahmassani, Major, and Khoury (2008), where persons’ perception of whether pharmaceutical therapies were effective in revealing or preventing their migraine attacks was connected to their sense of having internal locus of control. The present study further adds that having potent ways of handling attacks is a prerequisite for being able to focus on increasing one's general well-being, which in turn seems to affect positively the experience of living with migraine.

The persons who had reached an acceptance of migraine and viewed their disorder as a part of life had fewer feelings of guilt and took the attitude that they wanted to make the best of the situation. Those who had not reached peace of mind through acceptance struggled more to be able to function, as well as those who were not afflicted by migraine, and being unable to do so, they were more likely to see themselves as failures. This difference in outlook indicates that acceptance influences well-being. Acceptance has been shown to have relevance to the experience of migraine, irrespective of headache severity, by creating increased perceived control, a higher level of activity, and lower levels of pain-related interference (Chiros & O'Brien, 2011). The findings of the present study showed that the attitudes of the surrounding world, especially those of close friends, family, and influential people such as physicians, affected the person's inner sense of acceptance of being afflicted. Therefore, it is troublesome that previous research has shown that persons with migraine have experienced other people questioning the seriousness of their migraine and not been taken seriously by personnel in healthcare (Cottrell et al., 2002; Moloney et al., 2006; Rutberg & Öhrling, 2012). We suggest that more information about migraine can increase the understanding of living with it, and according to van Manen (1997), hermeneutic-phenomenological research can contribute to greater thoughtfulness and tactfulness towards others, although such a transformation requires an openness to change. Moreover, acceptance and commitment additive therapy among women with migraine has proven to be an effective treatment of headache disability, emotional distress, and the affective dimension of pain (Mo'Tamedi, Rezaiemaram, & Tavallaie, 2012). The findings of this study indicate that acceptance is an aspect that could be considered in the treatment of migraine in order to assist persons to feel well.

### Methodological considerations

The 19 persons who participated in this study had a wide range of experience of managing life to feel well while living with migraine. According to Norlyk and Harder (2010), the participants of a phenomenological study must have experience of the phenomenon under study, but it is not equally necessary to have a variation in age, gender, and background. One limitation of the present study could be that 11 of the 19 persons with migraine were recruited by their physical therapist. Their participation in physical therapy could indicate that they were active in wanting to improve their health. To address this issue, we included another eight persons who were not actively participating in physical therapy, and we thereby strived to collect experiences of a broader variation. Another limitation was that we did not collect data on the participants’ use of premedication or their satisfaction of it. It is possible that the participation in physical therapy indicates that they are not using or are not satisfied with their premedication. This interpretation is enhanced by the findings of another study that some of the persons also participated in, showing that the strong motive for them to participate in physical therapy was to decrease their intake of medication or not being satisfied with their medication (Rutberg, Öhrling, & Kostenius, 2013).

To enhance the quality of this hermeneutic-phenomenological study and to maintain openness to the phenomenon under study, we formulated the research question driven by a sincere curiosity (van Manen, 1997). Furthermore, we held numerous discussions through the study and made notes about our preunderstanding and presumptions, for example, about managing life with migraine and how we ourselves manage life to feel well. By adopting Drews (2001) suggestions on revealing one's preunderstanding and assumptions, we became more aware of our beliefs, of our ways of viewing the phenomenon, and of some views that we had been unaware of. Even though there is always more than one possible interpretation in hermeneutic-phenomenological research, we hope that the interpretations that we have found to be the most probable in this study can encourage health professionals to act with thoughtfulness when meeting persons with migraine.

## Conclusions

Much of the earlier research dealing with the ways in which persons with migraine manage their lives has focused on preventive strategies. However, feeling well while living with migraine requires more than merely averting the threat of migraine. The findings of this study show that managing life to feel well while living with migraine involved striving to build a basic foundation of safeness, through handling triggers and the symptoms of migraine. Managing life to feel well also meant striving to amplify the good in life, by acting thoughtfully to increase one's energy and joy, as well as by finding peace with being afflicted with migraine as it meant finding a sanctuary from which migraine was easier to handle emotionally. Therefore, it is a necessity for persons to use both preventive and promotive strategies to feel well when living with migraine.
